# Risk factors for progression to bacteremia among patients with nosocomial carbapenem-resistant Acinetobacter baumannii pneumonia in the Intensive Care Unit

**DOI:** 10.1007/s10096-023-04668-9

**Published:** 2023-09-28

**Authors:** Haiming Niu, Xiaoqing Shen, Hongkai Liang, Guishen Wu, Shaoqing Cai, Qian Shen, Kouxing Zhang, Miaolian Chen, Jianwei Li

**Affiliations:** 1 Department of Critical Care Medicine, Zhongshan People’s Hospital, 2 Sunwen Dong Road, Zhongshan, 528400 People’s Republic of China; 2 Department of Critical Care Medicine, The Third Affiliated Hospital, Sun Yat-Sen University, Guangzhou, People’s Republic of China

**Keywords:** Carbapenem-resistant Acinetobacter baumannii (CR-AB), Nosocomial, Pneumonia, Bacteremia, Risk factors

## Abstract

Antibiotic-resistant *Acinetobacter baumannii* (*A. baumannii*) is a common cause of hospital-acquired infections. This study aimed to identify independent factors associated with progression from nosocomial pneumonia to bacteremia in patients infected with carbapenem-resistant *A. baumannii* (CR-AB). From 2019 to 2021, we conducted a retrospective anaylsis of the medical records of 159 nosocomial CR-AB pneumonia patients in our Intensive Care Unit (ICU). We employed both univariate and multivariable logistic regression models to identify factors associated with the progression of nosocomial CR-AB pneumonia to bacteremia. Among the 159 patients with nosocomial CR-AB pneumonia, 40 experienced progression to bacteremia and 38 died within 28 days following diagnosis. Patients who developed bacteremia had a significantly higher 28-day mortality rate compared to those without bloodstream infection (47.50% vs. 15.97%). Multivariable logistic regression revealed that higher levels of C-Reactive protein (CRP) (OR = 1.01) and the use of continuous veno-venous hemofiltration (CVVH) treatment (OR = 2.93) were independently associated with an elevated risk of developing bacteremia. Among patients who developed bloodstream infection, those who died within 28 days exhibited significantly higher level of interleukin-6 (IL-6), a greater frequency of antifungal drugs usage, and a longer duration of machanical ventilation compared to survivors. Furthermore, the use of antifungal drugs was the only factor that associated with 28-day mortality (OR = 4.70). In ICU patients with central venous catheters who have CR-AB pneumonia and are on mechanical ventilation, higher CRP levels and CVVH treatment are risk factors for developing bacteremia. Among patients with bacteremia, the use of antifungal drugs is associated with 28-day mortality.

## Introduction

*Acinetobacter baumannii* (*A. baumannii*) is a non-fermentative Gram-negative aerobic coccobacillus and an opportunistic pathogen [1]. The widespread use of antibiotics has led to the emergence of various antibiotic-resistant A. baumannii strains [2], These include carbapenem-resistant *A. baumannii* (CR-AB) [3], multi-drug resistant *A. baumannii* (MDR-AB) [4] and pan-resistant *A. baumannii* (PDR-AB) [5]. The annual global incidence of *A. baumannii* infections is estimated to be approximately 1,000,000 cases, with 50% exhibiting resistance to multiple antibiotics, including carbapenems [6]. Currently, antibiotic-resistant *A.baumannii* has become a common cause of hospital-acquired infections, particularly in intensive care units (ICUs) [7, 8]. The antibiotic resistance of *A. baumannii* significantly limits treatment options [9, 10], leading to prolonged hospital stays and increased mortality among ICU patients [11].

*A. baumannii* infection primarily presents as pneumonia and bloodstream infections (bacteremia) [12]. Additionally, it can lead to urinary tract infections, post-neurosurgery meningitis, wound infections following trauma or surgery, and osteomyelitis [12]. A meta-analysis, comprising data from 27 worldwide studies, reported an overall mortality rate of 42.6% for *A. baumannii* causing hospital-acquired and ventilator-associated pneumonia [13]. Among *A. baumannii*-related infection, bloodstream infections is the most severe clinical type, with attributable mortality as high as 58.24% in cases of CR-AB bacteremia [14]. Several studies have explored risk factors associated with antibiotic- resistant *A. baumannii* infection, identifying factors such as prolonged hospital stay, current ICU admission, immunosuppression, advanced age, comorbidities, major trauma or burns, prior antibiotic use, invasive procedures, and indwelling catheterization or mechanical ventilation [15–23]. In a study by Kim et al. which analyzed 441 colonized patients in the ICU over a 7-year period, endotracheal intubation (odds ratio [OR], 5.88), ventilator support (OR, 3.70), and central venous catheterization (OR, 3.48) were identified as risk factors of bacteremia among patients colonized by MDR-AB [24]. However, little is known about the factors contributing to the progression from focal infection to bacteremia in patients infected with *A. baumannii*.

*A. baumannii* is a common pathogen causing nosocomial infections in ICUs in our hospital. However, despite similar clinical procedures being administered to all ICU patients, such as central venous catheterization and mechanical ventilation, we have observed that some patients infected with CR-AB are limited to pulmonary infections, while others are more prone to developing bacteremia. As a result, we hypothesized that, in addition to invasive procedures, there may be other risk factors contributing to bacteremia in CR-AB infected patients. Therefore, this study aimed to investigate the independent factors associated with progression from nosocomial pneumonia to bacteremia, as well as the mortality factors in CR-AB patients.

## Material and methods

### Study subjects

A retrospective study was conducted in a teaching hospital in southern China, equipped with a 62-bed intensive care unit (ICU). We retrospectively analyzed the medical records of patients diagnosed with nosocomial CR-AB pneumonia from January 2019 to December 2021.

We identified a total of 159 cases, which were divided into two groups for comparison, 40 cases with CR-AB pneumonia associated with bacteremia and 119 cases without bacteremia. The inclusion criteria were: 1) meeting the 2016 clinical practice guidelines by the Infectious Diseases Society of America and the American Thoracic Society for nosocomial pneumonia infection and bacteremia [25]; 2) detection of CR-AB through sputum or bronchoalveolar lavage culture conducted more than 48 h after ICU admission; 3) pneumonia-associated bacteremia, defined as the detection of an *A. baumannii* strain in peripheral blood culture for more than 48 h along with a positive sputum or bronchoalveolar lavage culture, excluding cases with positive transcatheter tip culture only, and no fever or systemic symptoms [26]; 4) aged ≥ 18 years. The exclusion criteria were: incomplete clinical data. This study was approved by the institutional review board of our hospital (Ethical Approval K2021-113). We obtained verbal informed consent from the patients themselves or the their immediate family through a telephone interview.

### Data collection

All data were collected from the medical records of 159 nosocomial CR-AB pneumonia patients. The baseline demographic and clinical characteristics included gender, age, APACHE II score at ICU admission, duration of ICU stay, and comorbidities such as hypertension, diabetes, coronary heart disease, chronic heart failure, cerebrovascular disease, chronic kidney disease, chronic liver disease, solid tumors, hematological diseases, and chronic obstructive pulmonary disease. 

The treatment-related data were recorded when CR-AB was detected in the sputum or bronchoalveolar lavage samples of the patients. These data included the use of carbapenem, antibiotics, antifungal therapy, acid suppressants, glucocorticoids, continuous veno-venous hemofiltration (CVVH), vasopressors, as well as the durations of mechanical ventilation and central venous catheterization.

The blood biochemical examination data were all recorded within 24 h of detecting CR-AB in the sputum or bronchoalveolar lavage of the patients. These data included white blood cell (WBC) count, neutrophilic granulocyte (NE) count, lymphocyte (L) count, platelet (PLT) count, procalcitonin (PCT) level, interleukin-6 (IL-6) level, C-reactive protein (CRP) level, albumin (ALB) level, prealbumin (PA) level.

### Statistical analysis

Continuous data were presented as mean ± standard deviation (SD) while categorical data were reported with number and percentage (%). For comparisons of means between groups, we used either the Student’s independent t-test or Mann-Whitney U test, depending on the normality assumption. Categorical data were assessed using the Chi-square test or Fisher’s exact test (if the expected value was ≤ 5).

We employed univariate and multivariable logistic regression models to investigate the association between independent variables and dichotomous outcomes, which included bloodstream infection and 28-day mortality. Independent variables found to be significant in the univariate analysis were entered into the multivariable model. Additionally, independent variables that remained significant in the multivariable model were identified as associated factors for the dichotomous outcomes. In cases of multicollinearity, we conducted correlation coefficient analyses, including Pearson’s correlation coefficient and point-biserial correlation coefficient, to explore the relationships among independent variables. We considered *p* ≤ 0.05 as indicating statistical significance for each test, with two-tailed analysis. All the analyses mentioned above were performed using IBM SPSS Version 25 (SPSS Statistics V25, IBM Corporation, Somers, New York).

## Results

### Patient clinical characteristics

**Fig. 1:**
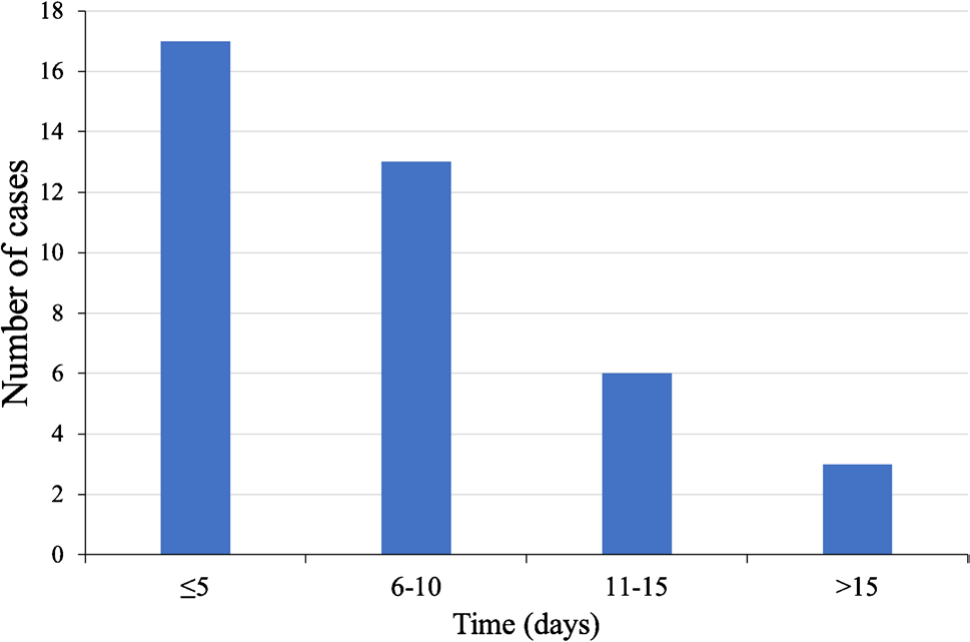
Time from positive sputum or bronchoalveolar lavage samples to bacteremia

A total of 159 nosocomial CR-AB pneumonia patients were included (mean age = 67.90 ± 14.54 years, 121 males and 38 females). The mean length of stay in the ICU was 25.21 ± 30.46 days, and the Apache II score was 23.62 ± 6.92. Among these patients, 40 developed pneumonia-associated bacteremia, with a mean time from positive sputum or bronchoalveolar lavage samples to bacteremia of 7.46 ± 6.24 days (Fig. 1).

Table 1 compares demographic and clinical characteristics between the two patient groups. The group with pneumonia-related bacteremia exhibited higher rates of CKD, CRP, PCT, IL-6 levels, and infection rates with other bacteria than the pneumonia-only group, while PA levels were lower (all *p* ≤ 0.05, Table 1). Additionally, patients with pneumonia-associated bacteremia had significantly higher rates of antifungal drug use, CVVH utilization, vasopressor administration, and a greater number of antibiotics, along with prolonged mechanical ventilation times (all *p* ≤ 0.05, Table 1).

**Table 1:** Demographic and clinical characteristics of CR-AB infected patients with or without bacteremia

	Bloodstream infection			
Parameters	No (*n* = 119)	Yes (*n* = 40)	All (*n* = 159)	*P*
Sex				0.833
Male	91 (76.47%)	30 (75.00%)	121 (76.10%)	
Female	28 (23.53%)	10 (25.00%)	38 (23.90%)	
Age, year	68.17 ± 15.06	67.10 ± 13.00	67.90 ± 14.54	0.689
Use of antibiotics	119 (100.00%)	40 (100.00%)	159 (100.00%)	1.000
Time from positive sputum or bronchoalveolar lavage culture to bacteremia, days	-	7.46 ± 6.24	-	-
ICU stay, days	25.62 ± 34.39	23.98 ± 13.42	25.21 ± 30.46	0.768
Apache II	23.69 ± 6.92	23.43 ± 7.01	23.62 ± 6.92	0.835
Comorbidities				
Hypertension	69 (57.98%)	22 (55.00%)	91 (57.23%)	0.741
Diabetes	31 (26.05%)	15 (37.50%)	46 (28.93%)	0.167
CHD	37 (37.00%)	16 (47.06%)	53 (39.55%)	0.300
CHF	48 (40.34%)	13 (32.50%)	61 (38.36%)	0.378
CVD	53 (44.54%)	18 (45.00%)	71 (44.65%)	0.959
CKD	31 (26.05%)	17 (42.50%)	48 (30.19%)	0.0499
CLD	12 (10.08%)	5 (12.50%)	17 (10.69%)	0.768
Solid tumor	30 (25.21%)	6 (15.00%)	36 (22.64%)	0.182
Hematological disease	8 (6.72%)	4 (10.00%)	12 (7.55%)	0.499
COPD	24 (20.17%)	7 (17.50%)	31 (19.50%)	0.713
Laboratory results				
CRP, mg/L	78.62 ± 70.67	140.10 ± 84.93	94.08 ± 78.91	< 0.001
PCT, ng/ml	7.00 ± 20.17	17.89 ± 26.46	9.74 ± 22.34	0.007
IL-6	523.48 ± 1168.36	1062.32 ± 1691.34	659.04 ± 1334.39	0.027
WBC, × 10^9/L	14.51 ± 7.32	13.90 ± 7.79	14.35 ± 7.42	0.655
NE, × 10^9/L	13.38 ± 9.36	12.44 ± 7.49	13.15 ± 8.91	0.565
L, × 10^9/L	0.87 ± 0.80	0.72 ± 0.51	0.83 ± 0.74	0.243
PLT, × 10^9/L	209.65 ± 123.71	200.38 ± 93.94	207.31 ± 116.72	0.665
ALB, g/L	32.36 ± 5.60	31.44 ± 5.72	32.13 ± 5.62	0.372
PA, mg/L	139.95 ± 77.94	113.13 ± 61.21	133.20 ± 74.82	0.049
Co-infection				
Other bacterial infection	72 (60.50%)	33 (82.50%)	105 (66.04%)	0.011
Fungalinfection	31 (26.05%)	16 (40.00%)	47 (29.56%)	0.094
Treatments				
Antifungal drugs	37 (31.09%)	21 (52.50%)	58 (36.48%)	0.015
Antacid	93 (78.15%)	36 (90.00%)	129 (81.13%)	0.098
Glucocoticoid	25 (21.01%)	9 (23.08%)	34 (21.52%)	0.785
CVVH	35 (29.41%)	24 (61.54%)	59 (37.34%)	< 0.001
Vasopressors	80 (67.23%)	33 (84.62%)	113 (71.52%)	0.037
Number of antibiotics				0.013
0–1	32 (26.89%)	2 (5.00%)	34 (21.38%)	
2	51 (42.86%)	21 (52.50%)	72 (45.28%)	
> 2	36 (30.25%)	17 (42.50%)	53 (33.33%)	
Ventilation time, hour	290.34 ± 233.26	418.25 ± 297.93	322.52 ± 256.22	0.006

ALB, albumin; CHD, corollary heart disease; CHF, chronic heart failure; CKD, chronic kidney disease; CLD, chronic liver disease; COPD, chronic obstructive pulmonary disease; CRP, C-reactive protein; CVD, cerebrovascular disease; CVVH, continuous veno-venous hemofiltration; IL-6, interleukin-6; L, lymphocyte; NE, neutrophilicgranulocyte; PA, prealbumin; PCT, procalcitonin; PLT, platelet; WBC, white blood cell

### Independent factors associated with bloodstream infection in CR-AB patients

To investigate the factors associated withpneumonia-associated bacteremia, both univariate and multivariable logistic regression models were performed. As shown in Table 2, a higher level of CRP (OR = 1.010, 95% CI = 1.007 to 1.013; *p* ≤ 0.05) and the utilization of CVVH treatment (OR = 2.93, 95% CI = 1.13 to 7.59; *p* ≤ 0.05) were found to be associated with an elevated risk of bacteremia.

**Table 2:** Univariate and multivariable logistic regression results of independent variables to combining bacteremia

	Univariate		Multivariable	
Parameters	OR (95% CI)	*P*	OR (95% CI)	*P*
Sex				
Male	ref	-		
Female	1.08 (0.47 to 2.49)	0.850		
Age, year	0.99 (0.97 to 1.02)	0.687		
ICU stay, days	1.00 (0.98 to 1.01)	0.768		
Apache II	0.99 (0.94 to 1.05)	0.834		
Comorbidities				
Hypertension	0.89 (0.43 to 1.82)	0.742		
Diabetes	1.70 (0.80 to 3.64)	0.170		
CHD	1.51 (0.69 to 3.32)	0.302		
CHF	0.71 (0.33 to 1.52)	0.379		
CVD	1.02 (0.50 to 2.09)	0.959		
CKD	2.10 (0.99 to 4.44)	0.052		
CLD	1.27 (0.42 to 3.87)	0.669		
Solid tumor	0.52 (0.20 to 1.37)	0.187		
Hematological disease	1.54 (0.44 to 5.42)	0.500		
COPD	0.84 (0.33 to 2.13)	0.713		
Laboratory results				
CRP, mg/L	1.010 (1.005 to 1.015)	< 0.001	1.010 (1.007 to 1.013)	0.021
PCT, ng/ml	1.02 (1.00 to 1.03)	0.013	1.01 (0.98 to 1.03)	0.663
IL-6	1.0003 (1.00002 to 1.001)	0.033	1.00 (1.00 to 1.00)	0.539
WBC, × 10^9/L	0.99 (0.94 to 1.04)	0.653		
NE, × 10^9/L	0.99 (0.94 to 1.03)	0.563		
L, × 10^9/L	0.65 (0.32 to 1.33)	0.238		
PLT, × 10^9/L	0.999 (0.996 to 1.002)	0.663		
ALB, g/L	0.97 (0.91 to 1.04)	0.370		
PA, mg/L	0.99 (0.99 to 1.00)	0.052		
Co-infection				
Other bacterial infection	3.08 (1.26 to 7.53)	0.014	1.86 (0.67 to 5.19)	0.234
Fungal infection	1.89 (0.89 to 4.02)	0.097		
Treatments				
Antifungal drugs	2.45 (1.18 to 5.09)	0.016	1.31 (0.53 to 3.20)	0.556
Antacid	2.52 (0.82 to 7.72)	0.107		
Glucocoticoid	1.13 (0.47 to 2.68)	0.785		
CVVH	3.84 (1.80 to 8.18)	< 0.001	2.93 (1.13 to 7.59)	0.027
Vasopressors	2.68 (1.04 to 6.94)	0.042	0.99 (0.30 to 3.28)	0.984
Number of antibiotics		0.034		0.189
0–1	ref	-	ref	-
2	6.59 (1.45 to 30.01)	0.015	3.94 (0.78 to 19.96)	0.098
> 2	7.56 (1.62 to 35.26)	0.010	2.38 (0.40 to 14.04)	0.338
Ventilation time, hour	1.002 (1.0004 to 1.003)	0.009	1.00 (1.00 to 1.00)	0.247

ALB, albumin; CHD, corollary heart disease; CHF, chronic heart failure; CKD, chronic kidney disease; CLD, chronic liver disease; COPD, chronic obstructive pulmonary disease; CRP, C-reactive protein; CVD, cerebrovascular disease; CVVH, continuous veno-venous hemofiltration; IL-6, interleukin-6; L, lymphocyte; NE, neutrophilicgranulocyte; PA, prealbumin; PCT, procalcitonin; PLT, platelet; WBC, white blood cell

The highest correlation coefficient among independent variables that were significant in univariate results was 0.60. Thus, no multicollinearity was observed.

### 28-day mortality

Out of the 159 nosocomial CR-AB pneumonia patients, 38 (23.90%) passed away within 28 days following the definitive diagnosis. The 28-day mortality rate was significantly higher among patients with pneumonia-associated bacteremia compared to those with pneumonia alone (47.50% vs. 15.97%, *p* ≤ 0.05).

### Independent factors associated with mortality in bloodstream-infected CR-AB patients

A subgroup analysis stratified by survival status among those with bacteremia demonstrated that patients who died within 28 days had a significantly higher level of IL-6, a higher rate of use of antifungal drugs, and a longer duration of mechanical ventilation (all *p* ≤ 0.05, Table 3).

**Table 3:** Clinical characteristics between patients who weresurvival or dead at 28-day within bacteremia group

	28 days after surgery		
Parameters	Survival (*n* = 21)	Dead (*n* = 19)	*P*
Sex			0.473
Male	17 (80.95%)	13 (68.42%)	
Female	4 (19.05%)	6 (31.58%)	
Age, year	64.43 ± 12.10	70.05 ± 13.63	0.175
Use of antibiotics	21 (100.00%)	19 (100.00%)	1.000
Time from positive sputum or bronchoalveolar lavage culture to bacteremia, days	6.14 ± 3.81	9.00 ± 8.08	0.157
ICU stay, days	20.38 ± 8.79	27.95 ± 16.51	0.075
Apache II	22.52 ± 8.06	24.42 ± 5.67	0.399
Comorbidities			
Hypertension	11 (52.38%)	11 (57.89%)	0.726
Diabetes	10 (47.62%)	5 (26.32%)	0.165
CHD	8 (47.06%)	8 (47.06%)	1.000
CHF	9 (42.86%)	4 (21.05%)	0.141
CVD	9 (42.86%)	9 (47.37%)	0.775
CKD	9 (42.86%)	8 (42.11%)	0.962
CLD	3 (14.29%)	2 (10.53%)	1.000
Solid tumor	3 (14.29%)	3 (15.79%)	1.000
Hematological disease	1 (4.76%)	3 (15.79%)	0.331
COPD	4 (19.05%)	3 (15.79%)	1.000
Laboratory results			
CRP, mg/L	132.29 ± 95.86	148.73 ± 72.57	0.548
PCT, ng/ml	17.71 ± 24.20	18.08 ± 29.44	0.965
IL-6	541.90 ± 1080.37	1637.51 ± 2058.14	0.039
WBC, × 10^9/L	14.41 ± 7.18	13.33 ± 8.57	0.666
NE, × 10^9/L	12.99 ± 7.01	11.83 ± 8.14	0.631
L, × 10^9/L	0.67 ± 0.41	0.77 ± 0.61	0.552
PLT, × 10^9/L	190.57 ± 83.82	211.21 ± 105.25	0.495
ALB, g/L	31.58 ± 5.51	31.29 ± 6.08	0.879
PA, mg/L	120.62 ± 63.28	104.84 ± 59.41	0.423
Co-infection			
Other bacterial infection	17 (80.95%)	16 (84.21%)	1.000
Fungal infection	8 (38.10%)	8 (42.11%)	0.796
Treatments			
Antifungal drugs	7 (33.33%)	14 (73.68%)	0.011
Antacid	17 (80.95%)	19 (100.00%)	0.108
Glucocoticoid	4 (20.00%)	5 (26.32%)	0.716
CVVH	10 (50.00%)	14 (73.68%)	0.129
Vasopressors	16 (80.00%)	17 (89.47%)	0.661
Number of antibiotics			0.152
0–1	1 (4.76%)	1 (5.26%)	
2	14 (66.67%)	7 (36.84%)	
> 2	6 (28.57%)	11 (57.89%)	
Ventilation time, hour	311.43 ± 203.12	536.32 ± 344.17	0.015

ALB, albumin; CHD, corollary heart disease; CHF, chronic heart failure; CKD, chronic kidney disease; CLD, chronic liver disease; COPD, chronic obstructive pulmonary disease; CRP, C-reactive protein; CVD, cerebrovascular disease; CVVH, continuous veno-venous hemofiltration; IL-6, interleukin-6; L, lymphocyte; NE, neutrophilicgranulocyte; PA, prealbumin; PCT, procalcitonin; PLT, platelet; WBC, white blood cell

To identify factors associated with 28-day mortality in bacteremia patients, both univariate and multivariable logistic regression models were performed. The results indicated that the use of antifungal drugs was associated with a higher 28-day mortality rate compared to patients without antifungal drug treatment (OR = 4.70, 95% CI = 1.11 to 19.95; *p* ≤ 0.05, Table 4).

**Table 4:** Univariate and multivariable logistic regression results of independent variables to 28-day mortality within bacteremia group

	Univariate		Multivariable	
Parameters	OR (95% CI)	*P*	OR (95% CI)	*P*
Sex				
Male	ref	-		
Female	1.96 (0.46 to 8.42)	0.365		
Age, year	1.04 (0.98 to 1.09)	0.174		
ICU stay, days	1.05 (0.99 to 1.10)	0.083		
Apache II	1.04 (0.95 to 1.14)	0.392		
Comorbidities				
Hypertension	1.25 (0.36 to 4.36)	0.726		
Diabetes	0.39 (0.10 to 1.49)	0.169		
CHD	1.00 (0.26 to 3.85)	1.000		
CHF	0.36 (0.09 to 1.44)	0.148		
CVD	1.20 (0.34 to 4.18)	0.775		
CKD	0.97 (0.28 to 3.40)	0.962		
CLD	0.71 (0.10 to 4.76)	0.721		
Solid tumor	1.12 (0.20 to 6.39)	0.894		
Hematological disease	3.75 (0.36 to 39.59)	0.272		
COPD	0.80 (0.15 to 4.13)	0.787		
Laboratory results				
CRP, mg/L	1.00 (0.99 to 1.01)	0.538		
PCT, ng/ml	1.00 (0.98 to 1.02)	0.964		
IL-6	1.00 (1.00 to 1.00)	0.061		
WBC, × 10^9/L	0.98 (0.90 to 1.07)	0.657		
NE, × 10^9/L	0.98 (0.90 to 1.07)	0.622		
L, × 10^9/L	1.48 (0.42 to 5.19)	0.543		
PLT, × 10^9/L	1.00 (1.00 to 1.01)	0.484		
ALB, g/L	0.99 (0.89 to 1.11)	0.875		
PA, mg/L	1.00 (0.99 to 1.01)	0.412		
Co-infection				
Other bacterial infection	1.25 (0.24 to 6.50)	0.787		
Fungal infection	1.18 (0.33 to 4.20)	0.796		
Treatments				
Antifungal drugs	5.60 (1.43 to 21.95)	0.013	4.70 (1.11 to 19.95)	0.036
Antacid	failed estimation	0.999		
Glucocoticoid	1.43 (0.32 to 6.39)	0.641		
CVVH	2.80 (0.73 to 10.75)	0.134		
Vasopressors	2.12 (0.34 to 13.24)	0.419		
Number of antibiotics		0.167		
0–1	ref	-		
2	0.50 (0.03 to 9.24)	0.641		
> 2	1.83 (0.10 to 34.85)	0.687		
Ventilation time, hour	1.00 (1.00 to 1.01)	0.031	1.00 (1.00 to 1.01)	0.063

ALB, albumin; CHD, corollary heart disease; CHF, chronic heart failure; CKD, chronic kidney disease; CLD, chronic liver disease; COPD, chronic obstructive pulmonary disease; CRP, C-reactive protein; CVD, cerebrovascular disease; CVVH, continuous veno-venous hemofiltration; IL-6, interleukin-6; L, lymphocyte; NE, neutrophilicgranulocyte; PA, prealbumin; PCT, procalcitonin; PLT, platelet; WBC, white blood cell

## Discussion

We conducted a retrospective study to investigate the independent factors associated with the progression from nosocomial pneumonia to pneumonia-related bacteremia in patients with CR-AB. Our study included a total of 159 ICU patients with CR-AB, of which 40 (25.15%) progressed to pneumonia-related bacteremia. This suggests that the progression from CR-AB pneumonia to pneumonia-related bacteremia is not uncommon. Furthermore, our findings indicate that patients with CR-AB pneumonia-related bacteremia have a significantly higher 28-day mortality rate when compared to the CR-AB pneumonia group, suggesting a worse clinical prognosis.

In previous studies, various risk factors for acquiring antibiotic-resistant *A. baumannii* bacteremia have been identified, including male gender, prior ICU stay, the use of cefoperazone–sulbactam, carbapenem, penicillins, endotracheal intubation, prior *A. baumannii* colonization, and cardiovascular failure [27–30]. In our univariate analysis, we identified risk factors for nosocomial CR-AB pneumonia-associated bacteremia, including the use of antibiotics, extended mechanical ventilation time, and bacteremia with other pathogens. Some of these findings were consistent with previous research [24, 31]. However, in the multivariable analysis, these variables did not reach significance, which may be attributed to the relatively small sample size and inconsistent characteristics of the included population, as tracheal intubation and central venous catheterization were performed in all patients in this study. In addition, discrepancies in patient characteristics and the variables controlled for in the multivariable analysis may also contribute to the differing findings between this study and previous ones.

In this study, CR-AB patients with pneumonia-associated bacteremia had a significantly higher level of CRP, a higher rate of CVVH treatment, and a longer ventilation time compared to those without bacteremia (all *p* ≤ 0.05). Furthermore, multivariable logistic regression analysis revealed that higher CRP level (OR = 1.01) and CVVH treatment (OR = 2.93) were associated with an increased risk of bacteremia. These variables have not previously been reported as factors for pneumonia-associated bacteremia in CR-AB patients. It's worth noting that CVVH treatment involves the placement of a vascular catheter, which, like other invasive procedures, can serve as a potential source of infection, thereby increasing the risk of bacteremia. CRP, on the other hand, serves as a marker of infections. In a study on bacteremia in community-acquired pneumonia, elevated CRP followed by lower plasma albumin was found to predict a higher risk of community-acquired bacteremia [32]. In addition, Ho et al. conducted a case-control study and found that CRP was highly specific for predicting bacteremia in critically ill patients [33]. In our study, the measurement of CRP and the time to diagnose pneumonia-related bacteremia differed from previous findings. On the one hand, this variation can be attributed to the limitations of retrospective studies, where blood cultures were not systematically collected; on the other hand, the diagnosis of bacteremia through the isolation of microorganisms from blood cultures proved to be a time-consuming process, with a positive reaction time ranging from 24 to 48 h. These two factors could contribute to delays in both CRP measurement and bacteremia diagnosis. CRP reflects the degree of inflammation in the body and can remain elevated for extended periods during ongoing inflammation. Higher CRP values indicated a more severe infection and predict a higher risk of progression from a focal to a systemic infection. This suggests that clinicians should monitor such patients more frequently and intervene early.

Several factors associated with mortality in patients with CR-AB infection have been reported, including old age, ICU stay after bacteremia, readmission within 90 days, tigecycline therapy, septic shock, multiple organ failure, a high Pitt bacteremia score, bacteremia following severe pneumonia, inappropriate empirical antimicrobial treatment, septic shock, chronic liver disease, chronic renal disease, hypertension, neutropenia, immunosuppressant use, and intubation [27, 30, 34]. These findings suggest that severity of baseline condition and inappropriate antibiotic therapy are the primary factors contributing to mortality in patients with CR-AB infections. In this study, among the 159 nosocomial CR-AB pneumonia patients in ICU, 38 (23.90%) patients died within 28 days after the definitive diagnosis. Notably, patients with bacteremia had a significantly higher 28-day mortality rate compared to those without bacteremia (47.50% vs. 15.97%), which is in agreement with the findings of Zhou et al. that bacteremia following severe pneumonia is a risk factor for MDR-AB bacteremia-related mortality [30]. Our results further revealed that among patients with pneumonia-associated bacteremia, those who died within 28 days exhibited significantly higher levels of IL-6, a higher rate of antifungal drug usage, and prolonged mechanical ventilation. The elevated IL-6 levels in bacteremia patients may be attributed, in part, to the higher rate of chronic kidney disease in this group, as it is well-known that CKD patients tend to exhibit increased IL-6 production and decreased clearance [35]. Univariate analysis showed that the use of antifungal drugs and prolonged mechanical ventilation time were associated with 28-day mortality in CR-AB patients. However, multivariable logistic regression demonstrated that the use of antifungal drugs was the only factor that associated with 28-day mortality (OR = 4.70). In our study, most antifungal drugs were used empirically based on the 2016 guideline and assessment scale for ICU patients issued by the Infectious Diseases Society of America [36]. The relatively low rate of fungal infection detection might be attributed to the subtle clinical manifestations of some critically ill patients and -limitation of detection. Nonetheless, the use of antifungal drugs still serves as an indicator of infection severity of infection in patients. This association with mortality should be further validated through future prospective studies. Due to our limited data, we cannot exclude the influence of other factors, such as disease severity, disease progression, and individualized treatment differences. The most significant challenge in treating *A. baumannii* infection is MDR, which negatively impacts mortality, length of hospital stays, and healthcare costs. A matched-controlled study demostrated that MDR increased mortality from 13 to 34% and extended hospital stay and health care costs in patients with *A. baumannii* bacteremia [37]. Park et al. identified that underlying malignancy, the need for mechanical ventilation, and CR-AB infection as risk factors associated with higher mortality in patients with *A. baumannii* bacteremia [38]*.* However, given that all patients included in this study were infected with CR-AB and required mechanical ventilation, we couldn't assess the individual impact of carbapenem resistance and mechanical ventilation.

We acknowledge several limitations in this study. Firstly, it was single-center study with a relatively small sample size, which may impact the generalizability of the results. Secondly, the lack of systematic blood culture collection for all CR-AB pneumonia patients poses a risk of misclassification between the two groups. Lastly, although our study identify a relationship between CRP and the progression of CR-AB pneumonia to associated bacteremia, we could not analyze the impact of changes in indicators over time or disease evolution, etc. on the final outcome. Future validation through multicenter, large-sample size prospective studies is warranted.

## Conclusion

In summary, this study revealed that among ICU ventilated patients with a central venous catheter, who developed CR-AB pneumonia, a higher level of CRP and the use of CVVH treatment were identified as risk factors for nosocomial pneumonia-associated bacteremia. Additionally, among patients with nosocomial pneumonia-associated bacteremia, the use of antifungal drugs emerged as a factor that associated with 28-day mortality. These findings hold the potential to inform recommendations for preventive and therapeutic guidelines for patients with CR-AB infections.

## Data Availability

The datasets generated during the current study are available from the corresponding author on reasonable request.
